# Antiprotozoal Effect of Snake Venoms and Their Fractions: A Systematic Review

**DOI:** 10.3390/pathogens10121632

**Published:** 2021-12-16

**Authors:** Zainab U. Abdullahi, Salihu S. Musa, Daihai He, Umar M. Bello

**Affiliations:** 1 Department of Biological Sciences, Federal University Dutsinma, Katsina 821101, Nigeria; zainabdeedeeumar@gmail.com; 2 Department of Biological Sciences, Bayero University, Kano 700006, Nigeria; 3 Department of Applied Mathematics, The Hong Kong Polytechnic University, Hong Kong 999077, China; daihai.he@polyu.edu.hk; 4 Department of Mathematics, Kano University of Science and Technology, Wudil, Kano 713101, Nigeria; 5 Centre for Eye and Vision Research (CEVR) Limited, 17W Hong Kong Science Park, Hong Kong 999077, China; 6 Department of Physiotherapy, Yobe State University Teaching Hospital, Damaturu 620101, Nigeria

**Keywords:** antiprotozoal, snake venoms, venom fractions, systematic review

## Abstract

Background: Protozoal infection is a lingering public health issue of great concern, despite efforts to produce drugs and vaccines against it. Recent breakthrough research has discovered alternative antiprotozoal agents encompassing the use of snake venoms and their components to cure these infections. This study collated the existing literature to examine the antiprotozoal effect of snake venoms and their fractions. Methods: We conducted a systematic review following the PRISMA guidelines. The PubMed and Embase databases were searched from their inception until 13 October 2021. Articles were screened at the title, abstract and full-text phases. Some additional studies were obtained through the manual search process. Results: We identified 331 studies via the electronic database and manual searches, of which 55 reporting the antiprotozoal effect of snake venoms and their components were included in the review. Around 38% of studies examined the effect of whole crude venoms, and a similar percentage evaluated the effect of a proportion of enzymatic phospholipase A2 (PLA2). In particular, this review reports around 36 PLA2 activities and 29 snake crude venom activities. We also report the notable phenomenon of synergism with PLA2 isoforms of *Bothrops asper*. Importantly, limited attention has been given so far to the antiprotozoal efficacies of metalloproteinase, serine protease and three-finger toxins, although these venom components have been identified as significant components of the dominant venom families. Conclusion: This study highlights the impact of snake venoms and their fractions on controlling protozoal infections and suggests the need to examine further the effectiveness of other venom components, such as metalloproteinase, serine protease and three-finger toxins. Future research questions in this field must be redirected toward synergism in snake venom components, based on pharmacological usage and in the context of toxicology. Ascertaining the effects of snake venoms and their components on other protozoal species that have not yet been studied is imperative.

## 1. Introduction

The evolutionary origin of snake venom has been traced back to the Cenozoic era [1]. Snake venoms have been identified as one of the most well-characterized animal venoms, with complex compositions of toxic, pharmacologically active proteins and peptides [2]. When compared with the venoms of other animals such as scorpions, spiders and cone snails, snake venoms are considered advanced due to their vast array of larger proteins and peptides that possess medicinal and toxicological effects [3]. About 50–100 components in snake venoms are distributed in dominant and secondary families, presenting multiple proteins and peptide isoforms [4]. The dominant families include secreted phospholipases A2 (PLA2s), snake venom metalloproteases (SVMPs), snake venom serine proteases (SVSPs) and three-finger toxins (3FTXs) [4]. The secondary families comprise cysteine-rich secretory proteins, L-amino acid oxidases (LAAOs), Kunitz peptides, C-type lectins, disintegrins and natriuretic peptides [5]. 

The compositions of snake venom vary interspecifically and intraspecifically [4], with various influencing factors including age, gender, location, diet and season [1]. These factors influence the diversity of toxins and their multifunctionality, and they greatly impact anti-venom production and envenomation treatment [6]. The pharmacological potentials of snake venoms have been grouped into hemotoxic, neurotoxic and cytotoxic effects [7]. The major toxins involved have been identified as PLA2s, SVMPs, SVSPs and 3FTXs, either singly or in combination. They are equally responsible for the pharmacological effects in snakebite victims [8]. 

Protozoan diseases are also a significant public health issue of great concern worldwide, especially in developing countries, with children being the most vulnerable population [9]. Millions of individuals globally experience one or more parasitic infections annually, and most of the morbidity and mortality due to protozoan infections are attributed to trypanosomatid and other *Apicomplexan* parasites [10,11]. However, *Toxoplasma gondii* and *Trichomonas vaginalis* are medically important intestinal protozoa [12]. Some of these are considered to be neglected tropical diseases, a term used to describe a group of chronic, debilitating and usually stigmatized conditions that mostly affect poor communities in tropical and subtropical regions [13]. 

The epidemiological control of protozoan diseases is still not satisfactory, due to the difficulties in achieving vector and reservoir control [1,12]. Equally, efforts toward developing vaccines against these persistent diseases are slow and not yet fruitful. Currently, chemotherapy remains the best choice for clinical management and disease control programs in endemic regions [12]. The chemotherapy agents in use are outdated and not fully cost-effective. In recent decades, efforts have been diverted towards developing novel chemotherapy agents to treat infectious diseases, due to increased drug resistance and the recognition of novel and previously unnoticed infectious agents [14]. The use of snake venoms and their components is an interesting and important concept, due to their potential as therapeutic molecules with antimicrobial features that could be used against shielded bacteria, fungi, parasites, protozoa and viruses [1,15]. 

We present a comprehensive systematic review of the existing literature on the antiprotozoal effect of snake venoms and their components. Notably, the review is intended to highlight the unconfirmed potentials of various snake venoms and their fractions as alternative treatments for protozoal diseases.

## 2. Methods

### 2.1. Search Strategy and Study Screening Processes

The methodology and reporting of the systematic review were conducted following the preferred reporting items for systematic reviews and meta-analyses (PRISMA) guidelines [16]. Pertinent databases (PubMed and Embase) were searched from inception until 13 October 2021, without restricting the year of publication. Studies reporting the antiprotozoal effect of snake venoms or their components were searched and included in the review. A comprehensive literature search was conducted for papers published in English, and the search terms across the two databases were “antiprotozoal OR antiprotozoal activity OR antiprotozoal drugs OR antiprotozoal agents” and “agents OR contrast agent” and “snake venom OR snake venoms OR snake venom metalloproteinase OR phospholipase PLA2 OR snake venom phospholipase A2 OR snake venom three-finger toxin OR snake venom serine proteinases”. The detailed search strategy adopted in this study is presented in Supplementary Table S1. Furthermore, the reference lists of the studies included in the review via the electronic database search were manually searched to ensure wider literature coverage. Citations were managed using EndNote version X9.0 (Clarivate Analytics, Philadelphia, PA, USA), and duplicates were removed using the built-in “find duplicates” feature. Two authors (Z.U.A. and S.S.M.) compiled the citations separately and later discussed them with the other authors to ensure an accurate literature report. Subsequently, the two authors independently and sequentially assessed the titles, abstracts and full text of the non-duplicated generated citations against the eligibility criteria of the study. Discrepancies in the outcomes of the screening processes between the two authors were resolved by further discussions and consultations with the other authors.

### 2.2. Study Selection and Eligibility Criteria

Articles were included in this review if they assessed the antiprotozoal effect of whole snake venoms or their fractions were published with at least an abstract in English. Restrictions were not placed on the study design; hence, studies of various designs (experimental, quasi-experimental, observational, case-control and case series, among others) were included. We excluded published reviews, review protocols, and conference abstracts. 

### 2.3. Data Extraction and Synthesis

To satisfy the set criteria of the review, two authors (Z.U.A. and S.S.M.) independently extracted all the relevant data using a pre-designed Excel sheet. The data extracted included the author details, date of publication, snake species under study, snake venom components or fractions, concentration of snake venom and components used, specificity of venoms and fractions to protozoan species, and snake venom and component-induced activity on protozoa. The extracted data were then compared, and cases of inconsistent outcomes were rectified via further deliberations among the authors. The data analysis followed the synthesis without meta-analysis (SWiM) guidelines [17]. 

## 3. Results

### 3.1. Literature Search Findings and Study Characteristics

**Figure 1:**
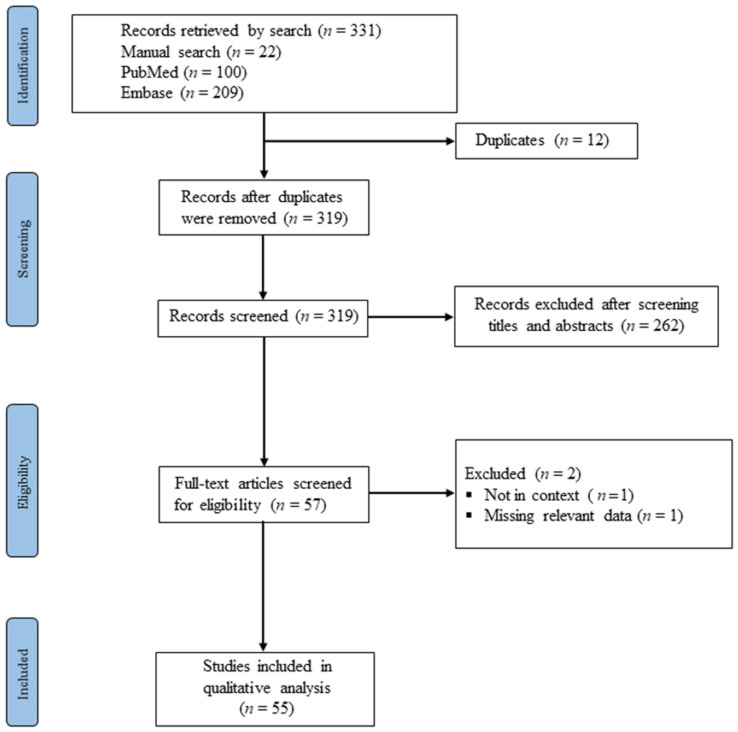
PRISMA diagram for the study search and selection processes.

The first search identified 309 articles through the electronic databases (100 in PubMed, and 209 in Embase). Additionally, 22 articles were added via the manual search of the reference lists of the included citations, totaling 331 papers (see Figure 1 for the outcomes of the search processes). After duplicates were removed, 319 studies remained. We screened the titles and abstracts of the 319 records against the stated eligibility criteria of the study. Finally, 55 studies were included for further synthesis and analysis. Figure 1 illustrates the flowchart of the study search and screening processes, and Table 1 presents the characteristics of the included studies.

### 3.2. Antiprotozoal Effect of Snake Crude Venoms

We identified 55 studies that met our inclusion criteria, of which 20 were conducted to evaluate the antiprotozoal efficacy of snake crude venoms [18,21,28,34,35,36,38,40,43,45,46,47,49,50,58,61,62,63,65,69]. We identified a previous study that reported the antiprotozoal effect of snake crude venoms, with some hypotheses about the specific snake venom fractions responsible for antiprotozoal activity without proof from laboratory trials [10]. Three reports [35,50,61] hypothesized that several proteins identified from the crude venom of *Bitis arietans* (*B. arietans*) could be responsible for its trypanocidal activity. Alape-Giron et al. [73] described snake venoms as a mixture of structured peptides, including enzymes and toxins, that comprise metalloproteases (41–44%), PLA2s (29–45%), serine proteases (4–18%), LAAOs (5–59%), disintegrins (1–2%), C-type lectin-like proteins (0.5%) and cysteine-rich secretory proteins (CRISPs; 0.1%). Similarly, Imam et al. [50] reported that the venom of *B. arietans* is composed of several catalytically active enzymes, including PLA2, LAAOs and CRISPs. Adade et al. [18] also reported that crovirin, a CRISP contained in the snake venom of *Crolatus viridis viridis,* showed promising activity against *T. cruzi*. PLA2s have equally been reported to have antitrypanosomal activity [34]. 

Furthermore, previous studies [28,69] showed the impact of the trypanocidal activity of LAAOs. However, this may exclude the possibility that the other proteins reported by Chechet et al. [35], which corresponded to those reported by Guidlolin et al. [74], were responsible for the antitrypanosomal activity, either singly or synergistically. According to Peichoto et al. [62], the activity of the crude venom on the protozoal species was due to trimorphin. However, several works [18,40,45,46] suggested the need for further research to ascertain which components possess antiprotozoal efficacy, though crucial information has been reported on them, including their molecular weight and thermal stability [45,46]. Similarly, a need was reported to further investigate the fractions of *B. jararaca* and *C.d. terrificus* with antigiardial potential, suggesting that more research will provide details on the mechanisms of action [75].

### 3.3. Antiprotozoal Effect of Snake Venom Components or Fractions 

LAAOs are oxidoreductase flavoenzymes that catalyze the stereospecific oxidative deamination of L-amino acids to produce the α-keto acids, NH_3_ and H_2_O_2_ [25]. They form part of several proteins in ophidians, particularly hemorrhagic venoms. LAAOs have been reported to possess the ability to induce apoptosis in several types of cells [25], including vascular endothelial cells, but the mechanism of action remains unclear. The LAAO activity has been proven to be due to H_2_O_2_ production, which, in turn, has been linked with the oxidation of several proteins in the plasma membrane [1]. Our systematic review found different documented antiprotozoal activities of LAAOs. Several researchers reported antileishmanial activity in the respective species [31,32,36,67,70]. Other [25,37,38,42,60] showed their influence on growth inhibition, cytotoxic activity, inhibitory effect, programmed cell death and parasite killing on trypanosomatids. Furthermore, the LAAOs of *Bothrops pirajai* resulted in maximal inhibition of infection with *T. gondii* [51].

PLA2s are enzymatic proteins with a low molecular weight. They are responsible for promoting hydrolysis of the 3-sn-phosphoglyceride-dependent calcium 2-acyl ester bond, resulting in lysophospholipids and fatty acid products [1]. The PLA2s of snake venoms may appear to be the same but could have different toxicological efficacies in their myotoxicity, neurotoxicity, anticoagulant activity, hemolysis, hyperalgesia, inflammation, edema, cytotoxicity, hypotension, and parasitic activity [10]. The activity of PLA2s on protozoal species varies across species of snakes and the protozoal organisms involved, as described in Table 1. Previous reports [34,47,71] indicated the inhibitory effects of PLA2s of the respective snake venoms on *P. falciparum*. According to many other studies [21,58,59,68], various PLA2s inhibited the cellular viability of *Leishmania* species. In addition, Borges et al. [29] and Borges et al. [30] reported that PLA2s of *B. pauloensis* inhibited parasite adhesion, intracellular proliferation, parasite viability, intracellular proliferation and pro-inflammatory cytokine production in T. *gondii*. Furthermore, the PLA2s of *B. pauloensis* induced in vitro cell death in *L. mexicana* [52], and Zieler et al. [72] reported that the PLA2s of *C. adamanteus* blocked the ookinete adhesion and oocyst formation of both *P. gallinaceum* and *P. falciparum*. According to a previous study [63], crotoxin B and its complex from *C. durissus cumanensis* exerted a cytotoxic effect against the mononuclear cells of *P. falciparum*, and another [19] reported that the crovirin from *C. viridis* could inhibit and lyse human-infective trypanosome species, including the intracellular amastigotes. However, despite the successful antiprotozoal activities of PLA2s on protozoal species, Costa-Torres et al. [38] reported that the PLA2s of *B. marajoensis* did not promote any inhibition of *L. amazonensis* or *L. chagasi* growth. Similarly, Grabner et al. [47] reported that the PLA2s of *B. marajoensis* did not promote the in vitro inhibition of cellular viability in *T. cruzi epimastigote*, even at 100 μg/mL.

Snake venom metalloproteases (SVMPs) are zinc-dependent proteinases of around 20–110 kDa [76]. They are grouped into P-I, P-II and P-III classes according to their structural domains. These toxins are significant in viper venom compositions and have a substantial role in the toxicity of these venoms. The origin of SVMPs is linked to disintegrin and metalloproteinase (ADAM) proteins, particularly ADAM28 [77], with the P-III class being the most basal structural variant, comprising metalloproteinase, disintegrin-like, and cysteine-rich domains. Subsequently, P-II SVMPs came from P-IIIs and consisted of a metalloprotease and disintegrin domain, with the latter particularly found in venom as a proteolytically processed product [1]. The final class, PI SVMPs, which have only the metalloproteinase domain, evolved on multiple independent occasions in specific lineages due to the loss of the P-II disintegrin coding domain. SVMPs contribute extensively to the hemorrhagic and coagulopathy venom activities following bites by viperid snakes. Their isoform diversity often presents in their venom, likely facilitating synergistic effects such as a simultaneous action on multiple steps of the blood-clotting cascade [1]. Reports [27,52,54] showed the antiprotozoal activities of a metalloproteinase from the *Bothrops* species on *T. gondii*, and *P. falciparum*, which is one of the most threatening and widespread species.

## 4. Discussion

A total of 55 articles on the antiprotozoal effect of snake venoms and their components were identified through our systematic search of the existing literature. The majority were on the antiprotozoal efficacy of PLA2s. Over 70% of the snake species reported were vipers, with very few reports on the *Colubridae* species [78]. A significant proportion (around 20%) constituted species of the *Elapidae* family. PLA2s form a considerable component in the venoms of vipers and elapids [78], due to their biomedical importance over others [79]. PLA2s have catalytically active and inactive components. Asp49-PLA2s are the catalytically active component, and Lys49-PLA2s are the catalytically inactive component, which can facilitate pharmacological effects regardless of catalytic activity [80,81]. Findings on both the catalytically active and catalytically inactive PLA2s were reported in our study. The mediation of antiprotozoal effects by PLA2s could occur through the interaction of either PLA2 phospholipids or PLA2 proteins. Interestingly, the commonly described receptors in the cell membranes are the vascular endothelial growth factor receptor-2 (VEGFR-2), M-type receptors, and nucleolin [82,83]. Bregge-Silva et al. [31] reported synergism involving the PLA2 isoforms of *B*. *asper*, which resulted in around a 10-fold increase in antiplasmodial activity during the association of AS49-PLA2 and LYS49-PLA2. 

Synergism is an important phenomenon that occurs in snake venoms, leading to evolving strategies to potentiate toxicities. Synergism exists between toxins or toxin complexes in various snake venoms, with PLA2s (toxins or subunits) the primary enablers [84]. Snake venoms can induce considerable toxicity, which may be due to many toxins’ cumulative or synergistic roles. Their compositions function together, directly or indirectly, and result in improved toxicity and pharmacological efficacy. Most synergisms of toxins have been noticed where SVSPs, PLA2s, 3FTxs and SVMPs were co-administered [84]. Synergism involving two PLA2s in *B. asper* has also been reported [85]. The ASP49-PLA2 and LYS49-PLA2 homologs were reported to have acted synergistically, leading to an increase in Ca^2+^ ions in the plasma membrane, in turn resulting in the rapid death of myotubes. Another study reported a synergistic phenomenon between the myotoxins of ASP49-PLA2 and LYS49-PLA2, which resulted in irreversible membrane and overall cell damage [86].

Concerning the antiprotozoal activity of whole crude venoms, variations in their activity and composition are not uncommon, leading to their unique potentials in biomedical research [79]. The past literature has noted that variations in snake venoms’ biochemical makeup occur even among closely related species and within species [87,88,89]. For instance, in pit vipers and adders, intra-genus or intra-specific variation in venoms has been documented [87,90]. These diversities are attributed to diet [87,91,92,93] or topography [94,95]. Other attributable factors include repetitions in toxin-encoding genes, production processes [96,97,98,99,100], and functional and structural diversifications [75,88,101,102]. For example, venom from *Laticauda semifasciata* (a sea snake) does not have a complex composition, and it has just two prominent families of proteins, 3FTxs and PLA2s. However, the venoms of rattlesnakes and mambas can have 50–100 peptides or proteins, representing around 10–20 protein families [84]. Generally, the predominant protein families in snake venoms significantly comprise phylogenetic trends. The venoms of cobras, kraits, mambas and hydrophids in particular have more negligible toxins, such as 3FTxs and PLA2s. In contrast, viperid venoms are made up of more significant fractions with enzymatic activities such as snake venom metalloproteinase and snake venom serine protease [84]. For instance, the venom of *C. durissus terrificus* is composed of amino acids, small peptides, carbohydrates, lipids, biogenic amines, and enzymes, whereas that of *B. jararaca* has peptides, serine, and metalloproteases as its constituents [75]. Hence, the activity of venoms varies with the difference in concentrations and compositions.

Aside from the role of snake species in the antiprotozoal effect, parasites also present contributing factors. Promastigotes and amastigotes are physiologically different in their sensitivity to drugs, with amastigotes having the greater capability to accumulate drugs [75]. Furthermore, Podešvová et al. [52] reported that variations in the compositions of parasite membranes could also be responsible for the differences in the activities of snake venoms and their fractions. Additionally, mechanisms including post-translational modifications, protein stability, and folding may likely influence toxin activity on parasites [52].

### 4.1. Strengths

This systematic review was conducted following an extensive literature search of the pertinent PubMed and Embase databases. Relevant citations were extracted using the reference lists of the included studies to ensure robust coverage of the existing literature. The systematic review covered studies on the antiprotozoal effect of crude venoms and their components from clinical studies and scientific reports. No restrictions were placed on the year of publication to ensure the thorough collation of relevant information. Equally, the study inclusion criteria were not restricted to snake species or components, to provide detailed information to the research community on the research question and the gaps in the literature.

### 4.2. Limitations

Despite the strengths of our systematic review, it has some limitations. First, we restricted inclusion to studies published in English, thereby limiting the ability to incorporate relevant data from studies in languages other than English. Additionally, incorporating a meta-analysis on the antiprotozoal efficacy of venoms and their fractions would have improved the quality of our work, which could be considered in future studies. 

## 5. Conclusions

This systematic review provides a general overview of the antiprotozoal effect of snake venoms and their components. We found varying antiprotozoal activities, presenting outstanding breakthroughs in the quest for alternative therapies for lingering protozoal infections. However, several variations were documented, including the concentrations of the crude venoms and fractions used, IC50 dosages, protozoan species, and antiprotozoal activities. These findings present challenges as to how the reviewed snake venoms and their fractions could serve as alternative antiprotozoal agents for many protozoal species, if not all. An excellent approach to this dilemma could be gearing research efforts toward understanding the relationships between venom components in the context of synergism, rather than toward studies on individual units, mainly because venomous snake species are numerous. Future studies also need to focus on other snake venom components that have received little attention. We recommend that other protozoan species should be subjected to trials with crude snake venoms and their fractions.

## Figures and Tables

**Table 1:** Features of the included studies.

Study	DOP	Snake Specie(s)	Venom/ Venom Fractions	IC_50_/ EC_50_/LD_50_ Dosages	Dosage Trial	Protozoans	Activity of Snake Venom/Snake Venom Fraction on Protozoal Species
Adade et al. [18]	July 2010	*Crolatus viridis viridis*	Crude venom	TCE: 0.5, 0.6, 0.7, 0.9, 1.0 μg/mL TTC: 0.3 μg/mL TCA: 0.075, 0.037, 0.29, 0.17 μg/mL (LD_50_)	0.25–500 μg/mL	*Trypanasoma cruzi*	Inhibited the cellular viability of *T. cruzi* epimastigote, trypomastigote, and amastigote. Exerted effect on the ultrastructure and intracellular survival of *T. cruzi.* About 76–93% reduction in the number of parasites was observed and up to 94–97% per cell after 9 h. However, at concentrations of 8–500 μg/mL, the venom could not promote antitrypanosomal activity.
Adade et al. [19]	October 2014	*Crolatus viridis viridis*	Crovirin	TCT: 1.10 ± 0.13 μg/mL (LD_50_) TCA: 1.84 ± 0.53 μg/mL (IC_50_) TBR:BSF; 2.06 ± 0.12 μg/mL (IC_50_) TBR: PCF: 1.13 ± 0.31 μg/mL(IC_50_)	TBR: BSF & PCF: 0.6–4.8; TCT: 0.45–4.8 μg/mL; TTCA: 0.45–3.6 μg/mL.	*T. cruzi* *T. brucie rodhesiense*	Active against all the human infective trypanosomatids including the intracellular amastigotes.

				LAAO: 1.21 ± 0.89 μg/mL; 1.05 μg/mL (IC_50_)	L.AP: 1.2–4.8 μg/mL;LAA:0.6–9.6 μg/mL	*L.amazonensis*	
Adade et al. [20]	August 2012	*C. viridis viridis*	PLA2	2.50 ± 1.42 mcg/mL 0.77 ± 0.5 mcg/mL (IC_50_)	0.3125–10 mcg/mL	*L. amazonensis*	Inhibited the parasites’ growth in vitro.
Alfonso et al. [21]	September 2019	*Bothrops mattogrossensis*	BmatTX-IV		62.4 μg/mL	*L. infantum,*	BmatTX-IV inhibited the cellular viability of *L. infantum* promastigotes in vitro and that of *T. cruzi* epimastigostes and also a cytotoxic effect on murine fibroblasts.
			Crude venom	L.P:11.9 μg/mL (IC_50_) TCE:13.8 μg/mL (IC_50)_	72.5 μg/mL	*T. cruzi*	
Allane et al. [22]	December 2018	*Cerastes cerastes*	Disintegrin_Cc	DTDR (IC_50_)	1 μg	*L. infantum*	Showed antileishmanial activity and severe morphological alterations of the *Leishmania* promastigotes.
Aranda-Souza et al. [23]	December 2018	*Bothrops leucurus*	BLL	1.5 ± 0.17 μg/mL and 1.3 ± 0.06 μg/mL (IC_50_) LAAO: 0.88 ± 0.24 μg/mL and 0.86 ± 0.07 μg/mL	0.01–3.3; 0.8 and 1.6 μM	*L. amazonensis L. braziliensis*	Inhibited promastigote growth and viability in both species through a mechanism that was dependent on galactose and calcium. Decreased the intracellular parasites. Caused severe changes in amastigotes, without apparent damage to the host cells. Increased the proinflammatory cytokines IL-6 and TNF-ð by infected macrophages in both species but with some variations in relation to IL-1ᵦ and IL-10.
Bandeira et al. [24]	December 2017	*Crotalus durissus terrificus*	Ctn, ctn (1–14), ctn (15–34), IL-37	Ctn TCE: Ctn(4.47 ± 0.9 μM); ctn (1–14):DTDRIC_50_; ctn (15–34):DTDRIC_50_	TCE &TCP:0.9–100 μM and TCA: 0.22 or 0.44 μM	*T. cruzi*	Ctn resulted in the inhibition of all *T. cruzi* developmental stages, including the amastigote, and showed a high selective index against trypomastigote. Cell death was induced by necrosis and morphological alterations.
				TCT: Ctn:0.22 μM ctn (1–14):33.1 ctn (15–34):9.5 μM			
Barbosa et al. [25]	January 2021	*Bothrops jararacuccu*	BjussuLAAO-II	DTDR IC_50_	1.56–12.5 μg/mL	*L. amazonensis* *L. braziliensis*	Both were severely cytotoxic to the two *Leishmania* spp., even at lower concentrations. However, at the same concentrations, both showed a different cytotoxic effect.
		*Bothrops moojeni*	BmooLAOO-II				
Barros et al. [26]	December 2015	*Crotalus durissus terrificus*	PLA_2_	LAP:52.07 μg/mL (IC_50_) Peptide fraction: 16.98 μg/mL (IC_50_)	0.5–2.5 μg/mL	*L. infantum*	Both showed in vitro leishmanicidal activity.
			Peptide fraction	LAAO: DTDR (IC_50_) Macrophages: PLA_2:98_ μg/mL; peptide:16.98 μg/mL	0.5–2.5 μg/mL		
Bastos et al. [27]	December 2008	*Bothrops neuwiedi*	Neuwiedase	BI: PI:7.70 μg/mL (IC_50_); PIR: 3.24 μg/mL AI: 4.84 μg/mL PIR: DTDR (IC_50_)	12 to 0.7 μg/mL	*T. gondi* infected human fibroblast	Inhibited the rate of infection by 71% and 61% following treatments before and after infection, respectively. The enzyme has the ability to degrade extracellular matrix components, which is necessary to sabotage *T. gondii* activity on infected cells.
Bhattacharya et al. [28]	September 2013	*Bungarus caeruleus*	Crude venom	L.P:14.5 μg/mL (IC_50_) L.A:11.2 μg/mL (IC_50_)	1–50 μg/mL	*L. donovani*	Showed antileishmanial activity against *L. donovani* in vivo and in vitro; the activity was partly mediated by an immunomodulatory activity that involved the macrophages.
Borges et al. [29]	September 2016	*Bothrops pauloensis*		DTDR IC_50_			The toxin showed reasonable cytotoxicity against HeLa cells at a higher concentration; however, the effect was reduced with a reduction in concentration. In addition, the toxin could not elicit effects on the viability of tachyzoites but lessened its adhesion and proliferation when the tachyzoites were treated before infection.
			BnSP-7		200–1.5 μg/mL	*Toxoplasma gondii*	
Borges et al. [30]	June 2018		Anti-BnSP-7 IgY antibodies		100–0.09 μg/mL	*Toxoplasma gondii* and *L. amazonensis*	Anti –BnSP-7 IgY antibodies reduced parasite viability and, at a concentration of 12.5 μg/mL, induced proliferation intracellularly.
Bregge-silva et al. [31]	December 2012	*Lachesis muta*	(L.A. A.O)	2.22 μg/mL (IC_50_)	0.5–32 μg/mL	*L. brasiliense*	Inhibited the activity of *L. brasiliense* promastigotes.
				DTDR IC_50_		*T. cruzi*	*T. cruzi* showed resistance.
Carone et al. [32]	October 2017	*Bothrops jararacussu*	BJussuLAAO-II	4.56 μg/mL (IC_50_)	0.5–32 μg/mL	*L. amazonensis*	Showed an antileishmanial and trypanocidal effect on promastigotes and amastigotes of *Leishmania* and *Trypanosome,* respectively.
				4.85 μg/mL (IC_50_)	0.93–50 μg/mL	*T. cruzi*	
Castanheira et al. [33]	March 2015	*Bothrops pauloensis*	BpLec	DTDR IC_50_	0.195–12.5 μg/mL	*T. gondii*	Reduced *T.gondi* parasitic activity after tachyzoite treatment.
Castillo et al. [34]	December 2012	*Bothrops asper*	Fraction V (containing catalytically active PLA_2_s)	1.42 ± 0.56 μg/mL (IC_50_)	25–200 μg/mL	*P. falciparum*	The whole venom and fractions showed activity against the parasite. Fraction V, however, had the highest toxicity compared to the whole venom and fraction VI.
			Fraction VI (containing a catalytically inactive PLA_2_-like protein)	22.89 ± 1.22 μg/mL (IC_50_)			
			Crude venom	0.13 ± 0.01 μg/mL (IC_50_)			
Chechet et al. [35]	December 2018	*Bitis arietans*	Crude venom	0.3085 μg/mL (IC_50_)	0.02–5.0 μg/mL	*T. brucei brucei*	Showed anti-trypanosomal activity by lysing the parasite across all different concentrations with little or mild lysis of the erythrocytes.
Ciscotto et al. [36]	March 2009	*Bothrops jararaca*	LAAO- active fraction &venom	DTDR IC_50_	0.8 mg/mL	*L. amazonensis*	The venom and LAOO-active fraction resulted in parasite viability of 69% and 47%, respectively.
Costa et al. [37]	September 2015	*Calloselasma rhodostoma*	CR-LAOO	L.C.P:16.66 μg/mL (IC_50_) L.B.P:24.47 μg/mL (IC_50_)	0.5, 2, 8, 32 μg/mL	*L. braziliensis*, *L. chagasi*	Caused cytotoxic effect on *T. cruzi* and *Leishmania* spp. promastigotes; the activity against all the trypanosomatids was significantly inhibited by catalase.
					0.5, 2, 8, 32 μg/mL	*L. infantum*	
					0.5, 2, 8, 32 μg/mL	*T. cruzi*	
Costa-Torres et al. [38]	April 2010	*Bothrops marajoensis*	PLA2 (BmarPLA2)	DTDR IC_50_	0.39–6.25 µg/mL	*L. amazonensis* and *L. chagasi*	For BmarPLA2, the dosage used (100 μg/mL) could not reach IC_50_; BmarTV and Bmar LAAO inhibited the growth of *L. amazonensis* and *L. chagasi* stages.
			Crude venom (BmarTV)	LAP:86.56 and LCP:79.02 μg/mL (IC_50_)	12.5–200 µg/mL		
			BmarLAAO	LAP:2.55 µg/mL and LCP:2.86 µg/mL (IC_50_)	0.39–6.25 µg/mL		
De Barros et al. [39]	July 2016	*Bothrops jaracussu*	PLA2	14.36 μg/mL (IC_50_)	100 μg/mL–6.25 μg/mL	*L. amazonensis*	Showed antileishmanial effect, reduced the promastigotes by 78%, and strengthened the macrophages’ viability by 82%. After 48 h, an amastigote reduction of up to 55% was recorded.
de Menezes et al. [40]	January 2012	*Bothropoides lutzi*	Crude venom	61.2 μg/mL (IC_50_)	6.25–200 μg/mL	*Leishmania chagasi*	Caused inhibitory effects on *L. amazonensis* and *L. chagasi* promastigotes. Repressed the growth of *T. cruzi* epimastigotes.
				234.6 μg/mL (IC_50_)	6.25–200 μg/mL	*L. amazonensis*	
				DTDR IC_50_	6.25–100 μg/mL	*T. cruzi*	
Dematei et al. [41]	June 2021	*Bothrops atrox*	BatxC,	4.90 (EC_50_)	0 to 50 μM; BatxC (0.70, 0.47, 0.23 μM); BatxC (C-2.14Phe) des-Phe (1.94, 0.97 and 0.48 μM) BatxC (C-2.15Phe) (0.93, 0.47 and 0.23 μM)	*L. amazonensis*	All showed antileishmanial activity on promastigotes and amastigotes and also induced morphological changes.
			BatxC (C-2.15Phe)	6.74 (EC_50_),			
			BatxC (C-2.14Phe) des-Phe1	8.86 μM (EC_50_)			
Deolindo et al. [42]	November 2010	*Bothrops jararaca*	LAAO	4.3 μg/mL (LD_50_)	10–60 μg/mL	*T. cruzi*	Induced antitrypanosomal activity, resulting in changes similar to those observed in programmed cell death. The activity was, however, reversed not only by the presence but also by the absence of a hydrophobic amino acid that was required for the process.
Deolindo et al. [43]	February 2005	*Bothrops jararaca*	Crude venom	10 μg/mL (IC_50_)	5, 10, 25, 50 μg/mL	*T. cruzi*	Both induced the programmed death of cells in *T. cruzi* epimastigotes, with the activity of crude venom being due to stress, through a process similar to that of apoptosis in metazoans.
El Chamy Maluf et al. [44]	April 2016	*Crotalus durissus*	Crotamine	1.87 μM (IC_50_)	1.25–20 μM	*P. falciparum*	Inhibited the development of *P. falciparum* in a dose-dependent pattern.
Fernandez et al. [45]	August 1994	*Cerastes cerastes* *Naja haje* *Vipera lebetina*	Crude venom	DTDR (IC_50_)	1–100 μg/mL	*T. cruzi, L. donovani infantum*	The venoms of *C. cerastes* and *N. haje* showed a growth inhibition effect on the trypanosomatids.
Gonçalves et al. [46]	March 2002	*Bothrops jararaca*	Crude venom	DTDR (IC_50_) K_0.5_: 0.1–0.3 μg/mL (IC_50_)	50 μg/mL	*L. major*	Resulted in ultrastructural alteration and inhibited the growth of *L. major* epimastigotes and amastigotes. Resulted in the ultrastructural alteration and inhibition in the growth of *T. cruzi* trypomastigotes.
					0.1, 1, 10, 100 μg/mL	*T. cruzi*	
Grabner et al. [47]	September 2017	*Bothrops marajoensis*	Crude venom: 0.14 ± 0.08μ g/mL (IC_50_) BmajPLA_2_-II(b): 6.41 ± 0.64 μg/mL (IC_50_)		Venom: 3–0.093 μg/mL; PLA2: 10–0.3125 μg/mL	*P. falciparum*	Showed antiplasmodial activity against the parasites.
			DTDR(IC_50_)		125 μg/mL	*T. cruzi*	Showed activity against the stages of trypanosome.
			BmajPLA_2_-II(b) Dosage used (100 μg/mL) did not reach IC_50_		100–6.25 μg/mL	*L. infantum*	Showed activity against the stages of *Leishmania.*
Guillaume et al. [48]	March 2004	*Najamossambica*	PLA_2_	2.3 pM (IC_50_)		*P. falciparum*	All the tested PLA2s inhibited the intraerythrocytic development of *P. falciparum.* All PLA_2_s showed toxicity against trophozoite as well as schizont stages.
		*Notechis scutatus*	Notexin	2.6 nM (IC_50_)			
		*Agkistrodon halys*	PLA_2_	82.3 pM (IC_50_)			
		*Vipera ammodytes*	Ammodytoxin A	2.8 nM (IC_50_)			
Hajialiani et al. [49]	April 2020	*Naja Naja Oxiana*	Venom fraction	0.026 μg/mL (IC_50_)	2.6 μg/mL–0.0000026 μg/mL	*P. falciparum*	The active fraction at the particularly stated concentration possessed anti-plasmodial efficacy.
Imam et al. [50]	February 2021	*Naja nigricolis*	Crude venom	0.411 μg/mL (IC_50_)	1.2, 2.4, 3.6 μg/mL	*Trichomonas vaginalis*	Showed trichomonicidal potency.
		*Bitis arietans*		0.805 μg/mL (IC_50_)			
Izidora et al. [51]	May/June 2011	*Bothrops pijarai*	BpirLAAO-I	BI: 1.83 μg/mL (ID_50_); 3.14 μg/mL AI: 1.20 μg/mL (ID_50_)	20 to 0.3 μg/mL	*T. gondii* in human foreskin fibroblasts	The enzyme was effective in inhibiting the infection of neighboring cells and, hence, the spread of the parasite, instead of targeting the primary infection and arresting parasite replication.
Kayano et al. [52]	November 2015	*Bothrops brazili*	Venom BbMP-1	Venom: 3.2 μg/mL (IC_50_) BbMP-1: 0.17 μg/mL (IC_50_)	20–0.001 μg/mLs	*P. falciparum*	Showed the biotechnological potential of the metalloproteinase as an antiplasmodial candidate.
Macedo et al. [53]	January 2015	*Crotalus durissis terrificus*	Crotamine, Crotamine in solution and in microparticles	DTDR IC_50_	100 to 3.1 μg/mL	*L. amazonensis* in infected macrophages	Caused a decrease in the number of amastigotes. When a comparison was made with its activity on infected macrophages; the biodegradable microparticles containing crotamine were trapped by macrophages, which led to an increase in TNF-α levels of about 196 pg/mL.
Martins et al. [54]	July 2019	*Bothrops moojeni*	Crude venom	0.5 ± 0.01 μg/mL (IC_50_)	20 μL	*P. falciparum*	Inhibited the growth of W2 strain *P. falciparum* in vitro.
			BmooMP α-I	16.14 ± 2.35 μg/mL (IC_50_)			
Mello et al. [55]	May 2017	*Bothrops atrox*	(BatxC)	TT: 0.44 μM (IC_50_) TE: 11.3 μM(IC_50_)	0.44–100 μm	*T. cruzi*	Inhibited all the developmental stages of *T. cruzi*, with a high selective index of 315, and also caused necrosis.
Mendes et al. [56]	December 2019	*Agkistrodon contortrix*	p-Acl	pAcl: L.A.P:50.98, (EC_50_) L.A.A: 57.23 (EC_50_) L.I.A: 220.32) μm (EC_50_)	L.A.P. & L.A.A:0–250 μm L.I.A:0–100 μm	*L. amazonensis*	Active against *L. amazonensis* and *L. infantum* promastigotes and amastigotes, with low cytotoxicity on primary murine macrophages.
			p-AclR7	27.19, 36.83, 70.72 μm (EC_50_)		*L. infantum*	
				P-ACLR7: 237.70 μM (CC_50_) P-Acl-232.88 μM (CC_50_)	0, 2.5, 50, 100, 150 μm	*L. infected* macrophages	
Merkel et al. [57]	September 2007	*Eristocophis macmahoni*	Spermine	186 ng/mL (IC_50_)	2–10 μg/mL	*T. brucie*	Caused autophagy in the parasite.
Moura et al. [58]	March 2014	*Bothrops mattogrossensis*	BmatTX-I	DTDR IC_50_	3.12, 6.25, 12.5, 25, 50, 100 μg/mL	*L. amazonensis*	Inhibited the cellular viability of *L. amazonensis* promastigotes in vitro.
			BmatTX-I II				
			BmatTX-I I				
			Crude venom				
Nunes et al. [59]	February 2013	*Bothrops pauloensis*	BnSP-7	LP:58.7 μg/mL (IC_50_) and LA:28.1 μg/mL (IC_50_)	L.P-25–200 μg/mL L.A-100 μg/mL	*L. amazonensis*	This resulted in the inhibition of parasite proliferation of the promastigotes and reduced the cellular viability of the amastigotes. The toxin also resulted in severe morphological changes in the promastigotes.
Paiva et al. [60]	May 2011	*Bothrops atrox*	BatroxLAAO	LDP: 4.3 μg/mL (EC_50_)	0.5–32 μM	*L.donovani*	Resulted in dose-dependent killing of the parasite.
				LMP: 4.5 μg/mL	0.5–32 μM	*L. major*	Resulted in the dose-dependent killing of *Leishmania* spp. promastigotes and *T. cruzi* trypomastigotes.
				LBP: 23.34 μg/mL (EC_50_)	0.5–32 μM	*L. braziliensis*	
				TCP: 62.8 μg/mL (IC_50)_	0.5–32 μM	*T. cruzi*	
Passero et al. [61]	July 2007	*Crotalus* species	*Crotalus durissus terrificus* (Cdt) venom	(4.70 ± 1.72 μg/mL IC_50_)	7.81–500 μg/mL		*Crotalus durissus terrificus* (Cdt) venom resulted in higher antileishmanial activity than Cdca. *Crotalus durissus cascavella* (Cdca) venom resulted in antileishmanial activity; however, a concentration of 44.30 μg/mL increased parasite numbers by 50%. Equally, the venom showed less antileishmanial activity at higher concentrations (281.00 μg/mL IC_50_).
			*Crotalus durissus cascavella* (Cdca) venom	(9.41 ±1.21 μg/mL IC_50_)			
			*Crotalus durissus collineatus* (Cdcol) venom	(281.00 ± 9.50μg/mL IC_50_)	7.81–500 μg/mL		
			Cdca crotamine-	19.95 ± 4.21 μg/mL (IC_50_)	3.12–100 μg/mL		
			Cdca crotoxin-	99.80 ± 2.21 μg/mL (IC_50_)			
			Cdca gyroxin-	3.80 ± 0.52 μg/mL (IC_50_)			
			Cdca convulvin	DTDR IC_50_			
Peichoto et al. [62]	July 2011	*Philodryas patagoniensis (PPV)*	Crude venoms	DTDR (IC_50_)	50.1–1.695 μg/mL	*L. major*	TblV showed significant antileishmanial activity at its highest concentration; however, it resulted in parasite proliferation at intermediate concentrations. PPV was not very active in inhibiting parasite growth and its highest concentration was required to inhibit 51.5% proliferation. PbV, PooV and HttV at their final concentrations did not significantly inhibit *L. major* growth. PLA2 (trimorphin) of TbLV caused a biphasic effect with potent cytotoxicity in a dose-dependent pattern and resulted in parasite proliferation at its highest concentration.
		*Philodryas baroni (PbV)*		DTDR (IC_50_)	438,524,562 μg/mL		
		*P. olfersi olfersi (POOV)*					
		*Hypsiglena torquata texana (HttV)*					
		*Trimorphodon biscutatus lambda (TlbV)*		(108.6 μg/mL IC_50_)	11.9–191 μg/mL		
		*(TblV)*	PLA2 (Trimorphin)	0.25 μM; 3.6 μg/mL	0.01–1 μM		
Quintana et al. [63]	November 2012	*Crotalus durissus* *cumanensis*	Crude venom	0.17 ± 0.03 μg/mL (IC_50_)	0.05–0.5 μg/mL	*P. falciparum*	The venom and the two fractions showed antiplasmodial activity against the mononuclear cells. Although all showed a cytotoxic effect, crotoxin B showed the highest at a concentration higher than the one required to exert an antiplasmodial effect.
			Crotoxin B	0.6 ± 0.04 μg/mL (IC_50_)	0.1–1.0 μg/mL		
			Crotoxin B complex	0.76 ± 0.17 μg/mL (IC_50_)	0.1–1.0 μg/mL		
				2.22 μg/mL (IC_50_)	0.5–2.00 μg/mL		
				DTDR IC_50_			
Sharifi et al. [64]	November 2021	*Naja Naja oxiana*	Venom fraction NNOV-FK	LTP: 46.59 ± 2.38 μg/mL:(IC_50_) LTA:0.18 ug/mL ± 0.02 (IC_50_) and L.IM: 0.51 μg/mL (IC_50_)	6.25–100 μg/mL	*L. tropica*	Showed severe leishmanicidal activity against developmental stages in a dimensional pattern. The Th1 indicators significantly improved (TNF-α, interleukins-12 and iNOS gene expression). Conversely, IL-10 (T helper 2 markers) were drastically reduced.
Shinohara et al. [65]	December 2005	*C. d. terrificus*	DTDR (IC_50_)	3.125–200 μg/mL		*Giardia duodenalis*	Both inhibited the growth of trophozoites, and the inhibition level varied with concentration and incubation times.
		*B.jararaca*		5–320 μg/mL			
Simoes-Silva et al. [66]	September 2021	*Bothrops asper*	Venom and acidic PLA2s; BasPAC-I, BASPAC-II, BASPAC-III, and BASPAC-IV and the basic PLA2s; BASPB-I, BASPB-II, BASPB-III, BASPB-IV and BASPB-V	8.6 μg/mL (IC_50_):	100–6.25 μg/mL	*L. infantum*	All the acidic, BASPAC-I, BASPAC-II, BASPAC-III, BASPAC-IV demonstrated action against *L. infantum* promastigotes and *T. cruzi* epimastigotes. The basic, BASPB-II, and BASP-IV showed activity against *P. falcifarum* with activity showing about a 10-fold increase when ASP49-PLA2 and LYS49-PLA2 were associated with each other, thereby proving a synergistic action between the PLA2 isoforms.
				34.7 μg/mL (IC_50_)	BASPB-II (100–6.25)	*T. cruzi*	
				BASPB-II:2.46; 0.98 μM (IC_50_) BASPB-IV: 0.019; 0.0019 μm (IC_50_)	BASPB-II:40–0.625 μm BASPB-IV (0.2–0.0031 μm)	*P. falciparum*	
Soares et al. [67]	July 2020	*Micrurus lemniscatus*	ML-LAAO	0.14 μg/mL (IC_50_)	5.0 to 0.03 μg/mL	*L. amazonensis*	Showed in vitro leishmanicidal action in a dose-dependent pattern, which was significantly reduced by catalase.
				0.039 μg/mL (IC_50_)		*L. chagasi*	
Stábeli et al. [68]	March/April 2006	*Bothrops moojeni*	MjTX-II	DTDR (IC_50_)	0.1–100 μg/mL	*L. donovani*	Inhibited the cellular viability of *L*. *amazonensis*, *L. braziliensis*, *L. donovani*, and *L. major* promastigotes in vitro.
						*L. major*	
				DTDR (IC_50_)		*L. braziliensis*	
						*L. amazonenis*	
Tempone et al. [69]	January 2001	*Bothrops moojeni*	Crude venom	Crude venom: LAP:7.56 ± 0.020 μg/mL (EC_50_)	30–0.15 μg/mL	*L. amazonensis* *L. chagasi* *L. panamensis*	Caused a killing effect in vitro against *Leishmania* spp., and activity was attributed to the activity of an enzyme that constitutes 1.5% of the venom, characterized as L-amino acid oxidase.
			LAOO	LAP: 1.44 ± 0.062 μg/mL LPP: 1.19 ± 0.0083 μg/mL (EC_50_) LCP: 1.08 ± 0.0024 μg/mL (EC_50_)	300–0.244 μg/mL		
Toyama et al. [70]	January 2006	*Crotalus durissus cascavella*	LAAO	2.39 μg/mL (IC_50_)	4.81–77 μg/mL	*L. amazonensis*	Resulted in severe antileishmanial activity on the *L. amazonensis* promastigote.
Vitorino et al. [71]	December 2020	*Bothrops diporus*	Enzymatically active PLA2s and homologs: BdTX-I,	2.44 μg/mL (IC_50_)	10–0.00488 μg/mL	*P. falciparum*	All the phospholipases showed antiparasitic activity against the *P. falciparum* W2 strain.
			BdTX-II	0.0153 μg/mL,			
			BdTX-III	0.59 μg/mL, respectively (IC_50_)			
Zieler et al. [72]	December 2001	*Crotalus adamanteus*	PLA_2_	DTDR IC_50_	0.0001–10 μmol L^−1^	*P. gallinaceum* and *P. falciparum*	Blocked ookinete adhesion and oocyst formation of *P. gallinaceum* and *P. falciparum*. Although PLA2 did not present a direct effect on the parasite, pretreatment of the midguts with its catalytically active or inactive form may strongly lessen the association between ookinete and midgut. This indicated that PLA2 functions by associating with the midgut surface and preventing the activity of ookinete in relation to it.

Abbreviations: P-Acl = *Agkistrodon contortrix* myotoxin; p-AclR7 = Acl homolog; Ctn = cathelicidin; Batxc = *Bothrops cathelicidin;* BLL = *Bothrops* *leucurus* lectin; Bplec = *Bothrops pauloensis* lectin; BPP = Bradykinin potentiate peptide; BSF = Blood Stream form, PCF = procyclic form; TCP: Tissue culture promastigotes; BatxC = Batroxicidin; MTx = Mojave toxin; MjTx-II = *Bothrops moojeni* myotoxin-II; BnSP-7 = *B. pauleonsis* toxin; BdTx = *B. diporus* toxin; BjTx = *B. moojeni* toxin; LAAO = L-amino acid oxidase; LIM *= Leishmania*-infected macrophages; NI = not indicated; BI = before infection; AI = after infection; IR = intracellular replication; PI = parasite invasion; ASA = all species above; LA = *Leishmania* amastigotes; L.P = *Leishmania* promastigotes; TT = *Trypanosome* trypomastigotes; DTDR IC50: dosage tried did not reach IC50; ADSC: at different serial concentrations; LAP: *L. amazonensis* promastigotes; LLP: *Leishmania panamensis* promastigotes; LCP: *Leishmania chagasi* promastigotes; LDP: *Leishmania donovani* promastigotes; LMP: *Leishmania major* promastigotes; LBP: *Leishmania braziliensis* promastigotes; TCT: *Trypasoma cruzi* trypomastigotes.ss.

## Data Availability

All data used in this study can be obtained from the public domain.

## Supplementary Materials

The following are available online at https://www.mdpi.com/article/10.3390/pathogens10121632/s1, Table S1: Search strategy.
