# Supplementary material for: Antiprotozoal Effect of Snake Venoms and Their Fractions: A Systematic Review
Source: Pathogens. 2021 Dec 16;10(12):1632. doi: 10.3390/pathogens10121632 (PMC8707848; doi:10.3390/pathogens10121632)
Supplement: Supplementary file 1 [file pathogens-10-01632-s001.zip › pathogens-1483866-supplementary.pdf]

Supplementary Materials

Table S1: Search strategy. This study focused on articles from the PUBMED and Embase in accordance with PRISMA guidelines for published research articles that reported the antiprotozoal efficacy of snake venoms and/ or their components. We searched all fields for antiprotozoal efficacy (search terms; antiprotozoal, antiprotozoal activity, antiprotozoal agent, agent, contrast agents, snake venom, snake venoms, snake venom metalloproteinase, Phospholipase PLA2, snake venom phospholipase A2, snake venom three-finger toxin, snake venom serine proteinase) being the being part of the most comprehensive online databases. Full search strategies for each database is given in **Table S1**.

Table S1. Search strategy.

Database	Number of studies	Search terms
PubMed	100	(“antiprotozoal” OR “antiprotozoal activity” OR “antiprotozoal drugs” OR “antiprotozoal agents”) AND (“agent” OR “contrast agents”) AND (“snake venom” OR “snake venoms” OR “snake venom metalloproteinase” OR “Phospholipase PLA2” OR “snake venom phospholipase A2” OR “snake venom three finger toxin” OR “snake venom serine proteinases”)
Embase	209	(‘antiprotozoal’ OR ‘antiprotozoal activity’ OR ‘antiprotozoal drugs’ OR ‘antiprotozoal agents’) AND (‘agent’ OR ‘contrast agents’) AND (‘snake venom’ OR ‘snake venoms’ OR ‘snake venom metalloproteinase’ OR ‘Phospholipase PLA2’ OR ‘snake venom phospholipase A2’ OR ‘snake venom three finger toxin’ OR ‘snake venom serine proteinases’)
Manual Search	22	Via references of the included studies
